# Primary prevention for offspring of parents with mental illness

**DOI:** 10.4103/0019-5545.70970

**Published:** 2010

**Authors:** T. S. Sathyanarayana Rao, Chittaranjan Andrade

**Affiliations:** Department of Psychiatry, JSS Medical College Hospital, Mysore, India; 1 Department of Psychopharmacology, National Institute of Mental Health and Neurosciences, Bangalore, India

Much literature has addressed caregiver burden in relation to depression,[1] anxiety disorders,[2] bipolar disorder,[3] schizophrenia,[4] dementia,[5] and other mental illnesses.[6] How are families, specifically children, affected when the patients are themselves the caregivers? This question is important because the offspring of parents with mental illness are at increased risk of medical, psychological, and social adversity for psychosocial and biological reasons, such as inadequate parental care, socioeconomic difficulties, genetic disadvantage, and psychotropic drug exposure in utero and during the puerperium.

Much literature is available on certain of these issues, such as the effects on children resulting from the use of psychotropic medications during pregnancy and lactation. However, there is only a modest body of research on the medical health of these children during their preschool years. Two very recent studies on the early survival of children of parents with mental illness are briefly reviewed. One study addresses the sudden infant death syndrome (SIDS) in infants of parents with a history of psychiatric admission, and the other addresses the natural and unnatural causes of death in preschool children of parents with mental illness. Both studies highlight new areas for primary prevention; areas that would otherwise be neglected in routine clinical practice.

## SUDDEN INFANT DEATH SYNDROME

Sudden infant death syndrome (SIDS) refers to sudden, unexplained death occurring during the first year of life. The risk of SIDS is known to be higher in infants of persons with mental illness.[7,8] Webb *et al*,[9] investigated the possible reasons for this finding. The study was based on data collected on nearly 2.5 million singleton live births in Sweden between 1978 and 2004. There were 1531 cases of SIDS in the whole cohort, and the crude rate of SIDS was 0.6 per 1000 live births.

The study threw up several important findings. Relative to the infants of women who were never admitted for psychiatric treatment, the risk of SIDS was trebled in infants of mothers with a history of psychiatric admission (OR, 3.1; 95% CI, 2.6–3.8). The risk was particularly high in mothers admitted for alcohol or drug-related disorders (OR, 6.5; 95% CI, 4.9–8.7), and was also high in mothers admitted with other diagnoses (OR, 2.3; 95% CI, 1.8–2.9). Relative to the infants of control fathers, the risk of SIDS was more than doubled in infants of fathers with a history of psychiatric admission (OR, 2.5; 95% CI, 2.0–3.1). The risk was significantly elevated with alcohol and drug-related diagnoses (OR, 2.8; 95% CI, 2.1–3.8), as well as with other diagnoses (OR, 2.1; 95% CI, 1.5–3.0).

The risk of SIDS was elevated nearly seven-fold when both parents had a history of admission for any mental illness (OR, 6.8; 95% CI, 4.7–10.0). Again, the risk was higher in association with alcohol and drug-related admissions (OR, 9.5; 95% CI, 5.5–16.4) than with admissions for other diagnoses (OR, 5.4; 95% CI, 3.2–9.2). Curiously, the risks were not significant for parental affective and non-affective psychoses, specifically, but this could have been because the analyses were underpowered. Strikingly, the risk remained elevated even if the last maternal admission was five or more years before the infant’s birth.

After a national campaign to reduce SIDS, the risk factor prevalence (especially maternal antenatal smoking) remained high in parents with a history of mental illness, and therefore, the relative risk of SIDS increased. During 1992–2004, smoking and individual social adversity measures together accounted for approximately half of the excess risk associated with maternal psychiatric admission, whereas the risk associated with obstetric factors remained minimal.

## PRESCHOOL MORTALITY

Chen *et al*,[10] provided a good review of the previous research and presented the first Asian data on preschool mortality in children of parents with mental illness. These authors used three population-based data sets in Taiwan to identify 3166 children with ICD-9 schizophrenia or affective disorder in one or both parents. A companion cohort of 25,328 control children was matched 8:1 with the case children, by maternal age and year of delivery. The risk of death from birth to the age of five years was examined using survival analysis adjusted for sociodemographic confounders and maternal medical comorbidities.

On univariate analysis, case children were more likely than control children to be underweight (<2.5 kg) at birth and to be premature (gestational age at birth <37 weeks). Case mothers were more likely to be poor, less well-educated, unmarried, diabetic, and hypertensive. Case fathers were more likely to be older and less educated.

There were 54 (1.7%) deaths among case children and 155 (0.6%) deaths among control children. Maternal mental illness nearly trebled the risk of all-cause mortality during preschool years (HR, 2.9; 95% CI, 2.0–4.2). However, paternal mental illness did not significantly influence the risk (HR, 1.5; 95% CI, 0.8–2.6). Maternal mental illness more than doubled the risk of natural death during preschool years (HR, 2.3; 95% CI, 1.5–3.5). Again, paternal mental illness did not significantly influence this risk (HR, 1.0; 95% CI, 0.5–2.0). Maternal mental illness was associated with a nine-fold increased risk of unnatural death during preschool years (HR, 9.1; 95% CI, 4.0–21.0). Paternal mental illness was also associated with a substantially elevated risk (HR, 6.1; 95% CI, 2.2–16.3).

For male children, parental mental illness raised the risk of both natural and unnatural death. For female children, parental mental illness only (but substantially) raised the risk of unnatural death. The risk of all-cause death was increased for both sexes. Common natural causes of death included congenital abnormalities, perinatal problems, and SIDS. Unnatural causes of death were homicide and accident. The proportional mortality rates were 20.4% and 11.1% for death by homicide and accident, respectively, in the children of parents with severe mental illness.

## IMPLICATIONS FOR PRIMARY PREVENTION

The SIDS study[9] indicates that the risk of SIDS is increased in the infants of mothers or fathers who have a history of admission for psychiatric treatment. The risk is particularly high in parents whose admission is for an alcohol or drug-related problem and when both parents have a history of psychiatric admission. The risk remains elevated even five years after the psychiatric admission. Smoking and social adversity appear to mediate much of the risk. Smoking, here, is probably a proxy for other illness-related variables because it is hard to imagine why smoking itself would predispose to SIDS.

Thus, mental illness, especially in the context of social adversity, may be a vulnerability marker for parents who, for example, do not receive health education about the supine position in which their infants should sleep; or parents who are too stressed to act on such education. Mentally ill parents with infants should therefore be provided with Counselling about proper infant sleep position in order to reduce the risk of SIDS. This is probably the most important component of a primary prevention measure and, being simple, is very easily implemented. Counselling should also focus on encouraging parents to stop smoking and to stop using alcohol and drugs, as substance use and abuse could result in infant neglect. Counselling is particularly important when both parents suffer from mental illness.

The preschool mortality study[10] suggests that, in Asian preschool children, maternal schizophrenia or affective disorder increases the risk of death due to natural as well as unnatural causes; paternal schizophrenia or affective disorder increases only the risk of death due to unnatural causes. Girl children are particularly vulnerable to death due to unnatural causes; this could reflect negative Asian attitudes toward the girl child. There is therefore a strong need to counsel parents with mental illness about child-rearing practices, and to supervise the physical and psychosocial health of the children, especially the girl child.

Preventive medicine in the discipline of psychiatry is a relatively neglected field; when opportunities for intervention arise, these should be seized. In this regard, the studies of Webb *et al*,[9] and Chen *et al*,[10] indicate just two areas for intervention in a metaphorical galaxy of possibilities.
